# AI-Driven Trajectory Planning of Dentatron: A Compact 4-DOF Dental Robotic Manipulator

**DOI:** 10.3390/biomimetics10120803

**Published:** 2025-12-01

**Authors:** Amr Ahmed Azhari, Walaa Magdy Ahmed, Mohamed Fawzy El-Khatib, A. Abdellatif

**Affiliations:** 1 Department of Restorative Dentistry, Faculty of Dentistry, King Abdulaziz University, Jeddah 21589, Saudi Arabia; 2 Mechatronics and Robotics Engineering Department, Faculty of Engineering, Egyptian Russian University, Cairo 11829, Egypt; 3 Mechanical Engineering Department, Arab Academy for Science Technology and Maritime Transport, Sheraton Branch, Cairo 11757, Egypt

**Keywords:** dental health, robotic manipulators, robot kinematics, robot dynamics, trajectory planning control, system verification

## Abstract

Dental caries is one of the most widespread chronic infectious diseases for humans. It results in localized destruction of dental hard tissues and has negative impacts on systemic health. **Aims**: This study aims to design, model, and control a novel 4-DOF dental robotic manipulator, Dentatron, specifically tailored for dental applications. The objectives were to (1) develop a compact robotic arm optimized for dental workspace constraints, (2) implement and compare three controllers—Computed Torque Control (CTC), Fuzzy Logic Control (FLC), and Neural Network Adaptive Control (NNAC), (3) evaluate tracking accuracy, transient response, and robustness in step and trajectory tasks, and (4) assess the potential of adaptive neural controllers for future clinical integration. **Materials and Methods**: The Dentatron system integrates a custom-designed robotic manipulator with adaptive controllers. The methodology consists of five main stages: robot modeling, control design, neural network adaptation, training, and evaluation. Simulations were performed to evaluate performance across joint tracking and Cartesian trajectory tasks using MATLAB 2022. Human-inspired trajectory design is fundamental to the Dentatron control and simulation framework to emulate the continuous curvature and minimum jerk characteristics of human upper-limb motion. The desired end-effector paths were formulated using fifth-degree polynomial trajectories that produce bell-shaped velocity profiles with gradual acceleration changes. **Results**: The study revealed that the Neural Network Adaptive Controller (NNAC) achieved the fastest convergence and lowest tracking error (<3 mm RMSE), consistently outperforming Fuzzy Logic Control (FLC) and Computed Torque Control (CTC). NNAC consistently provided precise joint tracking with minimal overshoot, while FLC ensured smoother but slower responses, and CTC exhibited large overshoot and persistent oscillations, requiring precise modeling to remain competitive. **Conclusion**: NNAC demonstrated the most robust and accurate control performance, highlighting its promise for safe, precise, and clinically adaptable robotic assistance in dentistry. Dentatron represents a step toward the development of compact dental robots capable of enhancing the precision and efficiency of future dental procedures.

## 1. Introduction

Dental health is a fundamental component of overall well-being, serving as both an indicator and contributor to systemic health [1,2]. Traditional methods for caries detection, such as visual–tactile examination and radiography, often exhibit limitations in sensitivity and specificity, hindering their effectiveness in determining the activity or progression of carious lesions. Advanced diagnostic techniques, including fiber-optic trans-illumination, quantitative light-induced fluorescence, laser fluorescence, electrical conductance measurements, digital radiography, optical coherence tomography, and intraoral scanners, provide more accurate information about carious lesions [3,4].

Human–robot collaboration (HRC) is increasingly being integrated into dentistry to improve surgical precision, reduce procedural time, and enhance patient safety. Recent advancements in robot-assisted dental procedures have demonstrated the potential of collaborative robotic systems in dental implant surgery, where robots assist in positioning, drilling, and ensuring the accurate placement of implants [5]. These robotic systems operate under human supervision and enhance procedural efficiency, minimizing surgical deviations and improving outcomes [6]. Moreover, semi-autonomous robotic platforms are being developed for oral surgery, utilizing monocular vision-based guidance to assist in delicate dental procedures while maintaining human oversight [7].

Beyond surgery, collaborative robots play a role in dental prosthetics, orthodontics, and automated diagnostics. AI-driven robotic systems assist in 3D scanning, treatment planning, and prosthesis fabrication, reducing manual workload and increasing accuracy [8]. Additionally, human–robot interaction (HRI) research in dentistry has focused on improving robotic adaptation to dental practitioners’ workflows, ensuring a smooth integration into clinical practice [9]. The success of robot-assisted procedures is closely linked to how effectively robots synchronize with human movements—a concept rooted in motor resonance—where the human perception of robot-assisted movements influences real-time collaboration [10]. As robotic technology in dentistry advances, further refinements in surgical planning, haptic feedback, and AI integration will enhance its applications in complex dental procedures.

For orthodontic treatments, digital orthodontics integrate machine learning algorithms with robotic arms to precise position brackets based on preoperative digital treatment plans. The incorporation of robotic technology in clear aligner production and orthodontic simulations is improving treatment predictability and reducing manual errors [11]. Robotic assistance is also emerging in endodontic microsurgeries and periodontal procedures, where high precision is required for root canal treatments and soft tissue management. Studies have explored the integration of micro-robotic surgical instruments into endodontic navigation systems, enabling minimally invasive root canal therapy with enhanced accuracy and reduced procedural time [12].

Another application for dental robots lies in the advancements in robot-assisted prosthodontics including the development of robotic crown lengthening surgery systems. These systems utilize robotic arms and AI-powered software to perform precise incisions and tooth adjustments, enhancing aesthetic and functional outcomes for prosthetic restorations [13]. AI-driven robotic platforms are also being used for automated tooth preparation and prosthesis fabrication, streamlining the process, and improving consistency in customized dental restorations [14]. On the other hand, robotic dentistry is developing into remote procedures via teledentistry platforms, where robotic systems can be controlled remotely for diagnostic and treatment assistance. This is particularly useful in rural areas where access to dental professionals is limited, enabling remote-controlled robotic interventions for emergency cases [15].

**Figure 1:**
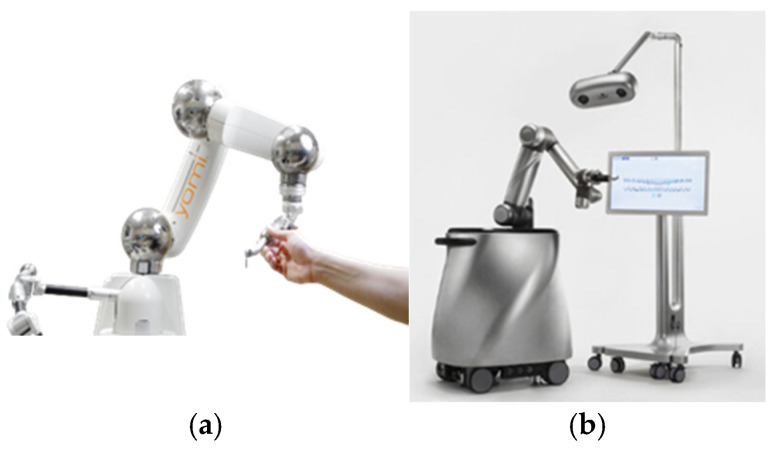
Examples of dental robots. (**a**) Yomi robot; (**b**) ADIR robot.

Examples of dental robots include Yomi, the first FDA-approved robotic system for dental implant surgery. Yomi assists in preoperative planning and real-time intraoperative guidance, ensuring greater accuracy in implant placement while allowing for dynamic adjustments based on patient-specific anatomical variations [16]. Research has demonstrated that Yomi improves surgical precision, reduces chair time, and minimizes the risk of complications compared to freehand implant placement methods [17]. Another example is The Autonomous Dental Implant Robotic (ADIR) system that has been developed to provide fully automated implant placement capabilities. Unlike Yomi, which operates under human supervision, ADIR is designed for the autonomous execution of implant drilling and placement, significantly reducing human intervention [18]. Studies indicate that ADIR offers high accuracy in edentulous patients, particularly in full-arch rehabilitation and flapless implant surgeries [19,20]. Both robotic dental systems are shown in Figure 1.

From previous examples, dental robots can be classified according to their mechanical manipulator, hardware configuration, type of perception, position/force controller, and level of autonomy [16]. Many examples of dental robots use off-shelf industrial manipulators with their integrated controllers. Dental robots like Theta [21], Dcarer [22], Remebot [23], and Yakebot [24] use Universal robot manipulators [25] as their main mechanical arm. This is somehow limiting the possibility of open-source upgrades in software and hardware. Additionally, these robots exhibit levels 1 and 2 of autonomy during operations.

In level 1 autonomy, the robot provides real-time guidance and positional constraints while requiring the operator to continuously control its movements. This collaborative framework allows the surgeon or dentist to retain full control, with the robot acting as an assistive mechanism to enhance precision and prevent deviations from predefined safe zones. An example of such a system is the Mako Smart Robotics platform (Stryker Corporation, Kalamazoo, MI, USA). In contrast, level 2 autonomy enables the robot to perform specific tasks independently based on discrete instructions from the operator and preprogrammed procedural steps. Here, the operator interacts with the system intermittently rather than continuously, allowing the robot to autonomously execute movements such as drilling or implant positioning.

From previous literature, the necessity for developing customizable and upgradable robotic manipulator systems with robust controllers for dental assistance was proven. To develop a surgical robotic assistant, the robotic arm must mimic human dentist kinematic movements during reaching tasks for object handling. Thus, for a robotic manipulator to be utilized, a robust position controller must be used to control these trajectories using the starting and end points as input data. These “biologically inspired” trajectories, referred to as “human-like,” are subsequently used for the trajectory planning of a serial robotic arm, which functions as a human substitute in our proposed collaboration scenario.

Several AI-based controllers are used for robotic manipulators. Adaptive Neural Network-Based Control (ANNBC) has been extensively applied to robotic manipulators to enhance their adaptability and performance in uncertain environments. The Adaptive Neural Network-Based Control (ANNBC) approach is designed to enhance the trajectory tracking performance of robotic manipulators by dynamically compensating for model uncertainties and external disturbances. Unlike traditional model-based controllers, which rely on the precise knowledge of system dynamics, ANNBC employs a neural network to approximate unknown nonlinearities in real-time.

Notable implementation is proposed by Tianli Li et al. [26], where they presented an ANN control scheme integrated with a disturbance observer to achieve precise trajectory tracking in robotic manipulators facing external disturbances and dynamic uncertainties. Another adaptive approach is presented by Yang et al. [27], who proposed an Adaptive Neural Network Control approach that utilizes dual neural networks to compensate for both kinematic and dynamic uncertainties. The controller is designed to quickly converge and achieve high accuracy, enhancing the manipulator’s adaptability and performance. Tien Pham et al. [28] proposed a controller that integrates neural networks (NNs) with dynamic surface control (DSC) to enhance tracking accuracy and stability. The Lyapunov-based adaptation ensures system stability while compensating for unknown system dynamics in real time.

Despite significant progress in dental robotics, current platforms face three major gaps. First, most existing systems (e.g., Yomi, ADIR, Theta, and Yakebot) are adapted from industrial robotic arms, limiting open-source customization and constraining clinical versatility. Second, many rely on preprogrammed guidance at autonomy levels 1 or 2, which restricts adaptability in dynamic clinical environments. Finally, robust controllers that can handle modeling uncertainties, patient variability, and real-time disturbances remain underexplored. These gaps underscore the need for compact, customizable dental robots equipped with adaptive AI-based control strategies.

In this paper, a new robotic manipulator is presented for dental care. It is a compact, versatile robot named “Dentatron”. The robot is custom designed, manufactured, and controlled for dental applications. For position control of its joints, two robust controllers are proposed, discussed, and compared. The first one is a model-based computer torque controller, where the mathematical model is derived and an adaptive component of the controller continuously estimates unknown parameters such as payload variations or friction effects, adjusting control efforts accordingly. However, its effectiveness relies on the accuracy of the dynamic model. The model-free adaptive control approach in this paper is a Neural Network Adaptive Controller artificial (NNAC) that dynamically learns the system behavior without requiring an explicit mathematical model for the robot.

The primary aim of this study is therefore to design, model, and control Dentatron as a novel 4-DOF dental robotic manipulator specifically tailored for dental applications. Two complementary control strategies are implemented and compared: a model-based Computed Torque Controller (CTC) and a model-free Neural Network Adaptive Controller (NNAC). In addition, a Fuzzy Logic Controller (FLC) is tested as a benchmark for smooth trajectory execution. In this work, biomimetic trajectories are motion profiles designed to mimic the smooth, coordinated, and physiologically natural movements found in human motor control. These trajectories were used as a unified reference for the CTC, FLC, and NNAC controllers, ensuring that all comparisons reflected tracking ability rather than differences in input signals. By imposing smooth, human-like motion profiles, the trajectory creates a realistic and clinically appropriate test that prevents abrupt movements in the dental workspace. This setup allows the simulations to fairly evaluate how effectively each controller reproduces dentist-like kinematics in terms of tracking error, overshoot, settling time, and overall motion quality. The objectives are fourfold: (1) to develop a compact and versatile robotic arm optimized for the geometric and ergonomic constraints of the dental workspace; (2) to implement and compare three advanced controllers—Computed Torque Control (CTC), Fuzzy Logic Control (FLC), and Neural Network Adaptive Control (NNAC)—under simulated dental tasks; (3) to evaluate tracking accuracy, transient response, and robustness across step and trajectory tasks; and (4) to assess the potential of adaptive neural controllers for future clinical integration in robotic dentistry.

## 2. Materials and Methods

### 2.1. Dentatron’s Mechanical Design, Kinematics, and Dynamics

#### 2.1.1. Overview of the Robotic Manipulator

**Figure 2:**
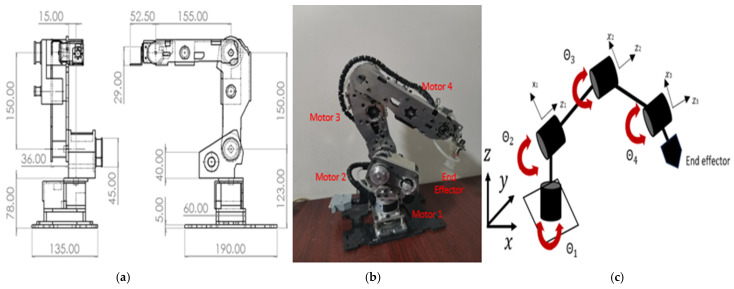
(**a**) Mechanical design of Dentatron robotic arm where the dimensions are in mm. (**b**) Real image of the manufactured robot. (**c**) The kinematic skeleton of the robot.

**Figure 3:**
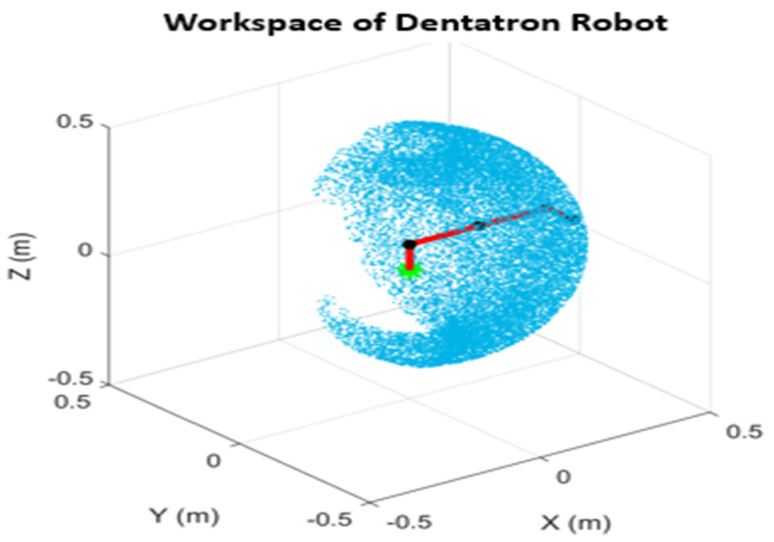
The workspace of Dentatron robotic arm.

The robotic manipulator utilized for this system is named Dentatron. It is an articulated robot with 4 DOFs. It is designed to be operated by DC motors with position and velocity feedback. Its end effector is to carry intraoral scanners and dental operation tools. Its maximum reachability is 357.5 mm, while its minimum reachability is 50 mm. The robot repeatability is ± 0.02 mm, and its payload is 1.5 kg. It has a relatively small footprint of 128 mm and a lightweight design at approximately 3.5 kg (excluding wires). The mechanical design of the robotic arm and its kinematic skeleton are shown in Figure 2. The robot workspace is presented in Figure 3.

#### 2.1.2. Robot Kinematics

To proceed with robot modeling and control, robot kinematics are explained in this section. The robot is modeled using a DH convention technique, as shown in Table 1.

The total transformation matrix elements of the robot are calculated according to the general form shown in Equation (1) as follows:

(1) $$T_{total}\;=\;\left[\begin{array}{cccc} R_{11} & R_{21} & R_{31} & P_{x}\\ R_{21} & R_{22} & R_{32} & P_{y}\\ R_{31} & R_{32} & R_{33} & P_{z}\\ 0 & 0 & 0 & 1 \end{array}\right]$$

where each cell is denoted as follows:

$$R_{11}=\cos\theta_{1}\cos\left(\theta_{2}+\theta_{3}+\theta_{4}\right)$$

$$R_{12}=-\cos\theta_{1}\sin\left(\theta_{2}+\theta_{3}+\theta_{4}\right)$$

$$R_{13}=\sin\theta_{1}$$

$$R_{21}=\sin\theta_{1}\cos\left(\theta_{2}+\theta_{3}+\theta_{4}\right)$$

$$R_{22}=-\sin\theta_{1}\sin\left(\theta_{2}+\theta_{3}+\theta_{4}\right)$$

$$R_{23}=-\cos\theta_{1}$$

$$R_{31}=\sin\left(\theta_{2}+\theta_{3}+\theta_{4}\right)$$

$$R_{32}=\cos\left(\theta_{2}+\theta_{3}+\theta_{4}\right)$$

$$R_{33}=0$$

$$P_{x}=-a_{2}\;\cos\theta_{1}\;\sin\theta_{2}\;+\;a_{3}\;\cos\theta_{1}\;\cos\left(\theta_{2}+\theta_{3}\right)\;+\;a_{4}\;\cos\theta_{1}\;\cos\left(\theta_{2}+\theta_{3}+\theta_{4}\right)\;+\;d_{2}\;\sin\theta_{1}$$

$$P_{y}=-a_{2}\sin\theta_{1}\sin\theta_{2}+a_{3}\sin\theta_{1}\cos\left(\theta_{2}+\theta_{3}\right)+a_{4}\sin\theta_{1}\cos\left(\theta_{2}+\theta_{3}+\theta_{4}\right)-d_{2}\cos\theta_{1}$$

$$P_{z}=a_{2}\cos\theta_{2}+a_{3}\sin\left(\theta_{2}+\theta_{3}\right)+a_{4}\sin\left(\theta_{2}+\theta_{3}+\theta_{4}\right)+d_{1}$$

The previous matrix is later used to calculate the robot inverse kinematic equations, which can be summarized as follows:

(2) $$\theta_{1}=atan2\left(R_{13},-R_{23}\right)$$

(3) $$\theta_{2}=\mathrm{atan}2\left(P_{y}^{\prime },P_{x}^{\prime }\right)-\mathrm{atan}2\left(a_{3}\sin\theta_{3},a_{2}+a_{3}\cos\theta_{3}\right)$$

(4) $$\theta_{3}=\mathrm{atan}2\left(\pm \sqrt{1-\cos^{2}\theta_{3}},\cos\theta_{3}\right)$$

(5) $$\theta_{4}=\mathrm{atan}2\left(R_{31},R_{32}\right)-\theta_{2}-\theta_{3}$$

#### 2.1.3. Trajectory Planning

After calculating the inverse kinematic model for the robot in the previous section, the robot must follow a Cartesian path as an end effector-based motion. This is achieved by trajectory planning. In this paper, we adopt a 5th degree polynomial trajectory to ensure smoothness and a limited error path, as can be seen in Equation (6):

(6) $$q_{d}\left(t\right)=a_{0}+a_{1}t+a_{2}t^{2}+a_{3}t^{3}+a_{4}t^{4}+a_{5}t^{5}$$

where $a_{0}$, $a_{1}$, …, $a_{5}$ are coefficients determined based on initial and final boundary conditions. For a fifth-order polynomial trajectory with boundary conditions

(7) $$q(0)=q_{0},q(T)=q_{f},\dot{q}(0)=0,\dot{q}(T)=0,\ddot{q}(0)=0,\ddot{q}(T)=0$$

The coefficients are given by

(8) $$a_{0}=q_{0},a_{1}=0,a_{2}=0,a_{3}=\frac{10(q_{f}-q_{0})}{T^{3}},a_{4}=-\frac{15(q_{f}-q_{0})}{T^{4}},a_{5}=\frac{6(q_{f}-q_{0})}{T^{5}}$$

The corresponding derivatives are

(9) $$\dot{q}\left(t\right)=3a_{3}t^{2}+4a_{4}t^{3}+5a_{5}t^{4}$$

(10) $$\ddot{q}\left(t\right)=6a_{3}t+12a_{4}t^{2}+20a_{5}t^{3}$$

Therefore, these equations ensure smooth position, velocity, and acceleration profiles.

### 2.2. Control Approaches

#### 2.2.1. Model-Based Adaptive Controller

The model-based adaptive controller is designed using the dynamic model of the Dentatron robotic arm, ensuring precise trajectory tracking by compensating for nonlinearities and external disturbances. The controller utilizes Computed Torque Control (CTC), where the robot dynamics are described by the following Equation (11). q is the joint position of the robot arm, while $\dot{q}$ and $\ddot{q}$ are the joint velocities and acceleration. $M\left(q\right)$, $C\left(q,\dot{q}\right)\dot{q}$, $G\left(q\right),$ and τ are the inertia matrix, Coriolis matrix, gravitational torque vector, and input control torque, respectively.

(11) $$M\left(q\right)\ddot{q}+C\left(q,\dot{q}\right)\dot{q}+G\left(q\right)=\tau$$

To achieve accurate trajectory tracking, a Computed Torque Control (CTC) approach is employed. The control laws are formulated in Equation (12) as follows:

(12) $$\tau =M\left(q\right)\ddot{q_{d}}+C\left(q,\dot{q}\right)\dot{q_{d}}+G\left(q\right)+K_{p}e+K_{d}\dot{e}$$

The desired trajectory is defined as $q_{d}$, with associated velocity $\dot{q_{d}}\;$ and acceleration $\ddot{q_{d}}$. The position and velocity tracking errors are denoted as e and $\dot{e}$, respectively. The controller gains $K_{p}$ and $K_{d}$ are the proportional and derivative gains, respectively.

**Figure 4:**
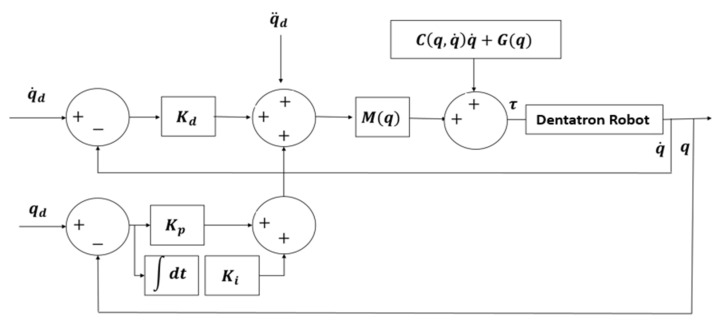
The block diagram describes the applied CTC on Dentatron robot.

To overcome uncertainties, an adaptive control law is introduced to the dynamic model, as shown in Equation (13):

(13) $$\dot{\hat{\theta }}=\Gamma Y^{T}e$$

where $\dot{\hat{\theta }}$ represents the estimated parameter vector, Γ is the adaptation gain matrix, while $Y(q,\dot{q})$ is the regression matrix derived from the robot dynamics. A block diagram that describes the implementation of the CTC is shown in Figure 4.

#### 2.2.2. Model-Free Controllers

**Figure 5:**
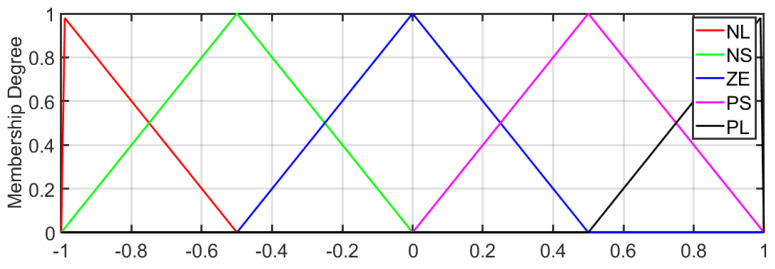
Normalized membership functions for error, change in error, and control action.

For the Dentatron robotic arm, two model-free controllers are used for position control. The first one is the Fuzzy Logic Controller (FLC). It is designed to control the joint motion of the Dentatron robot. The controller considers the position error and its rate of change to compute a suitable control signal. The controller fuzzification method is Mamdani, while the defuzzification method is centroid. The controller has five triangular membership functions (MFs): Negative Large (NL), Negative Small (NS), Zero (ZE), Positive Small (PS), and Positive Large (PL). The output variable is the control action (u) corresponding to the torque/command sent to the joint actuator. It is also defined in the normalized range [−1,1] [−1,1] [−1,1] with the same five membership functions (NL, NS, ZE, PS, and PL), ensuring consistency and smooth control signal generation. Figure 5 shows the input–output membership functions.

**Figure 6:**
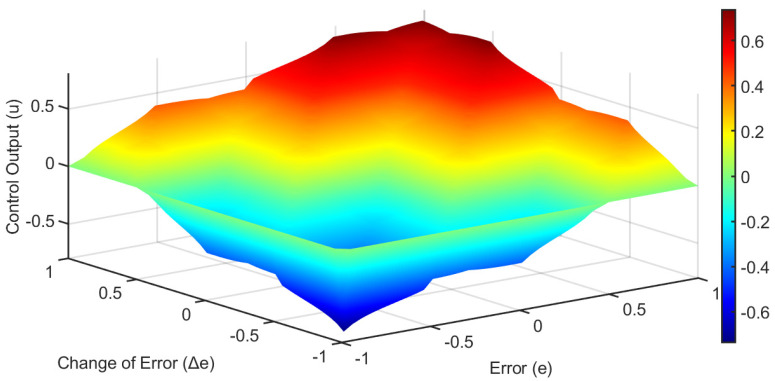
Fuzzy surface of the controller FLC applied on Dentatron robot model.

The rule base was designed using expert knowledge of robotic joint dynamics and consists of 25 fuzzy rules. The complete rule base is summarized in Table 2, and the fuzzy surface can be shown in Figure 6.

The second one is a Neural Network-Based Adaptive Controller (NNAC) implemented to dynamically adjust control inputs based on sensory feedback, eliminating the need for an explicit system model. The neural network approximates the nonlinear robot dynamics and generates appropriate control signals to ensure trajectory tracking.

Two special functions are added to the NNAC, which are the composite control law and Lyapunov constraint projection for bounded weight adaptation. This implementation provides robust performance under parametric uncertainties and non-modeled dynamics, which is ideal for high-precision dental applications.

The NNAC controller uses a compact feedforward neural network (FFNN) to learn and compensate for the Dentatron robot’s unknown nonlinear dynamics without requiring a system model. The network receives the joint positions and velocities as an 8-element input, passes them through tanh activation functions, and uses a linear weight matrix with 32 adaptive parameters to estimate the robot dynamics. These weights are updated online using a composite learning rule that combines tracking errors with prediction errors, enabling fast and reliable adaptation under uncertainties and disturbances. A Lyapunov-based projection operator keeps all weights within a safe range, ensuring stability during rapid learning. The final torque command blends the neural estimate with PD feedback, allowing the controller to achieve accurate trajectory tracking while remaining robust to nonlinearities, friction, and parameter variations.

The control objective is to ensure that the joint angle vector $q(t)\in R^{4}$ tracks a desired trajectory $q_{d}(t)\in R^{4}$, while relying solely on measured joint positions and velocities. The neural network input vector is defined by the concatenation of current joint positions and velocities as

(14) $$\phi \left(t\right)=\left[\begin{array}{c} q\left(t\right)\\ \dot{q}\left(t\right) \end{array}\right]\in R^{𝟠}$$

This vector is processed through a hyperbolic tangent activation function to produce the network basis functions as follows

(15) $$\sigma \left(\phi \right)=\tanh\left(\phi \right)\in R^{𝟠}$$

The network output estimates the unknown robot dynamics using a linear parameter approximation as follows

(16) $$\hat{f}\left(q,\hat{q}\right)=W^{⊤}\left(t\right)\sigma \left(\phi \left(t\right)\right)$$

where $W^{⊤}\left(t\right)\in R^{8\times 4}\;$ is the neural weight matrix adaptively updated at each timestep. The final control torque input $\tau \left(t\right)\in R^{4}\;$ is calculated as

(17) $$\tau \left(t\right)=\hat{f}\left(q,\hat{q}\right)+K_{p}e\left(t\right)+K_{d}\dot{e}\left(t\right)$$

While the tracking error terms are defined as

(18) $$e\left(t\right)=q_{d}\left(t\right)-q\left(t\right),\;\dot{e}\left(t\right)=\dot{q_{d}}\left(t\right)-\dot{q}\left(t\right)$$

The main innovation of this controller lies in the composite learning rule that fuses both tracking and model prediction errors for faster and more stable convergence. The model prediction error is computed as

(19) $$\hat{f}\left(t\right)=\tau \left(t\right)-\hat{f}\left(q,\hat{q}\right)$$

The previous rule is combined into the weight adaptation law as

(20) $$W\left(t\right)=-\Gamma \left(\sigma \left(\phi \left(t\right)\right)e^{⊤}\left(t\right)+\eta \sigma \left(\phi \left(t\right)\right)\hat{f^{⊤}}\left(t\right)\right)$$

where $\Gamma \in R^{8\times 8}$ is the adaptation gain matrix, and η > 0 is the prediction error scaling coefficient. Euler integration is used to update the weights:

(21) $$W\left(t+\Delta t\right)=W\left(t\right)+\dot{W}\left(t\right)\Delta t$$

To maintain Lyapunov stability and prevent weight exponential increase during rapid learning, the updated weights are projected into a predefined bounded domain using a hard projection operator:

(22) $$W_{ij}\left(t\right)=\;\left\{\begin{array}{c} W_{max}\;,{\;if\;W}_{ij}\left(t\right)>W_{max}\\ {-\;W}_{max},\;{if\;W}_{ij}\left(t\right)<-\;W_{max}\\ W_{ij}\left(t\right),otherwise \end{array}\right.$$

**Figure 7:**
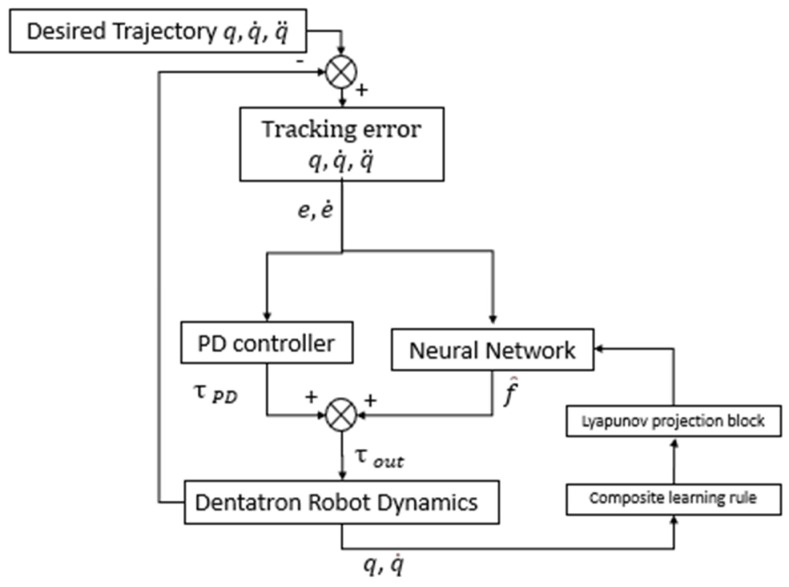
The block diagram describes the applied NNAC on Dentatron robot.

In our implementation, $W_{max}\;=\;10$, Γ = 5, and η = 0.5. This configuration allows the Dentatron robot to adaptively learn and reject unknown nonlinearities in its dynamics while ensuring stable trajectory tracking. A block diagram that describes the implementation of the NNAC is shown in Figure 7.

## 3. Results, Verification, and Discussion

The robot is modeled using the equations from Section 2 to calculate its inverse kinematics and inverse dynamics. To validate the proposed control approach, simulations were conducted using MATLAB for the Dentatron robotic arm. The robot joints were controlled using NNAC, FLC, and CTC controllers to test and evaluate its performance under two case studies. Both are displayed in the following subsections.

### 3.1. Joint Position Tracking

**Figure 8:**
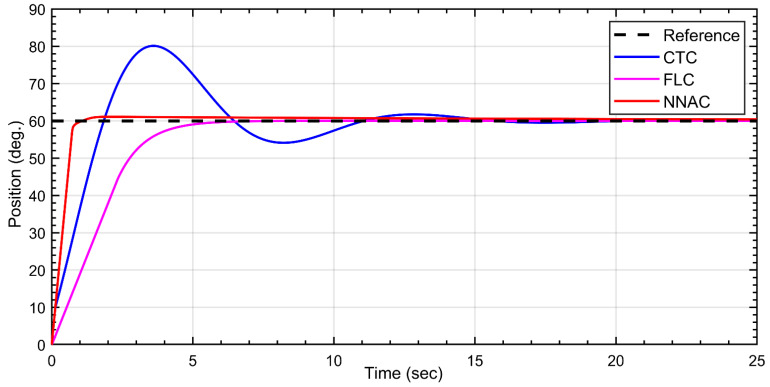
Tracking response of Link-1 under NNAC, fuzzy (FLC), and CTC controllers to a 60° step.

The Dentatron robot was subject to reference angles for all joints. For the first link, the desired trajectory is to 60 deg. As shown in Figure 8, all three controllers converge to the desired position but with distinct transient behavior. NNAC (red) reaches the setpoint fastest with a very small overshoot (≈1–2°) and short settling time. FLC (magenta) shows a slower, monotonic rise with virtually zero overshoots and a smooth, critically damped profile. CTC (green) is the most aggressive: it exhibits a pronounced overshoot (peaking around ~80°), followed by an undershoot (~55°) and damped oscillations before settling near 60°. Overall, NNAC offers the quickest accurate tracking, FLC provides the smoothest/no-overshoot response, and CTC incurs the largest transient excursion.

**Figure 9:**
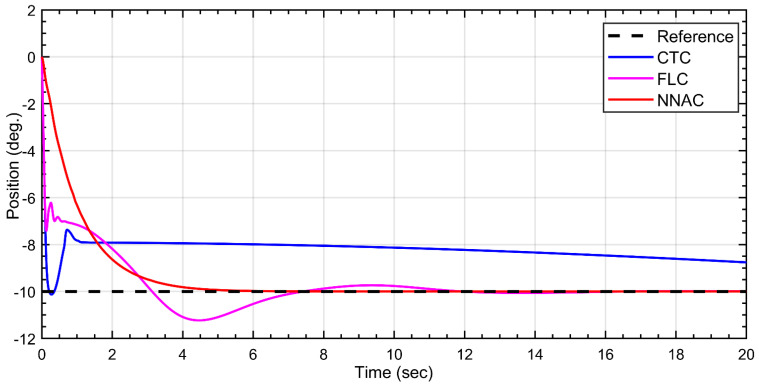
Tracking response of Link-2 under CTC, FLC, and NNAC controllers to a −10° step.

For the second link, all controllers move the link toward the −10° target but with different transients. NNAC (red) gives a smooth, monotonic decay with no overshoot and zero steady-state error, settling close to the setpoint within a few seconds. FLC (green) exhibits a noticeable overshoot (≈10–15%, down to about −11.5°) and a lightly damped oscillation before converging; it settles around 12–15 s near the target. CTC (magenta) reacts fastest initially but shows a brief undershoot/peaking and then maintains a residual offset (~1° at 20 s), indicating steady-state error. Overall, NNAC provides the most accurate and well-damped tracking, FLC converges with moderate overshoot, and CTC is quick but the least accurate at steady state, as shown in Figure 9.

**Figure 10:**
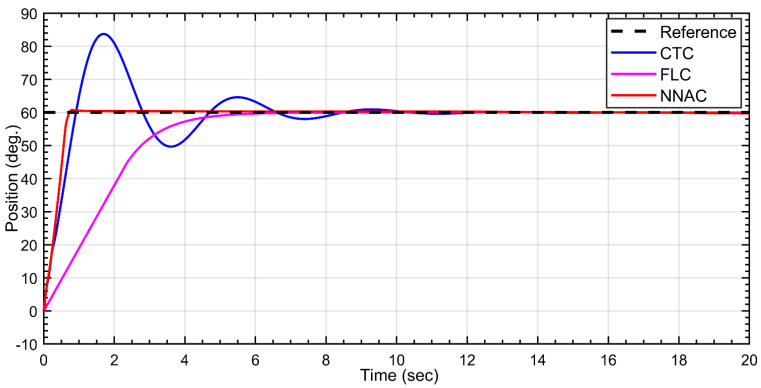
Tracking response of Link-3 under NNAC, CTC, and FLC to a 60° step.

For the third link, all controllers reach the setpoint but with different transients. NNAC (red) rises fastest and settles near 60° within ~1–1.5 s with negligible overshoot. CTC (magenta) is aggressive: it produces a large overshoot to ≈85° (~40% over), then an undershoot to ≈50°, followed by lightly damped oscillations that decay and settle around 10–12 s. FLC (green) is the smoothest but slowest, exhibiting a monotonic, no-overshoot rise that converges to 60° after ~7–9 s. Overall, NNAC delivers the quickest well-damped tracking, FLC prioritizes smoothness, and CTC incurs the largest transient excursions, as shown in Figure 10.

**Figure 11:**
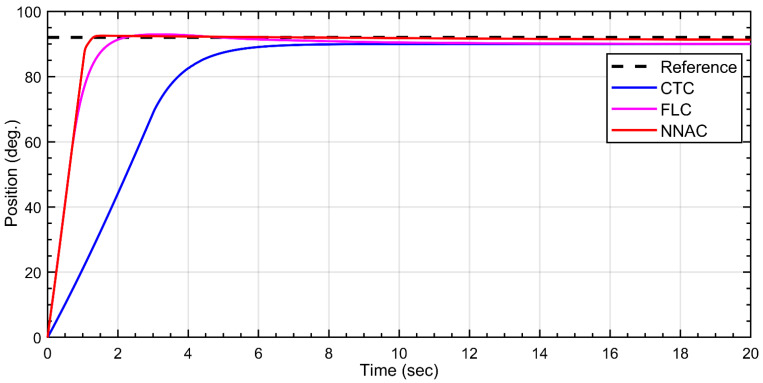
Tracking response of Link-4 under NNAC, FLC, and CTC controllers to a ≈90–92° step.

For the fourth link, all controllers reach the target angle with negligible steady-state error but differ in transient speed as shown in Figure 11. NNAC (red) achieves the fastest rise and settles in ≈2 s with only a very small overshoot (<~1–2°). FLC (green) is slightly slower, showing a minor overshoot followed by a short decay, settling in ≈3 s. CTC (magenta) is the slowest: it follows a near-linear ramp and reaches the neighborhood of the setpoint only after ≈6–8 s with no overshoot but the longest settling time. Overall, NNAC provides the quickest well-damped tracking, FLC is close with mild overshoot, and CTC is the most sluggish.

### 3.2. Robot Trajectory Tracking

To evaluate the performance of the dental robot arm in executing a predefined 3D trajectory, a sequence of five waypoints was programmed to guide the end effector through a path in Cartesian space. The trajectory was designed to simulate controlled motion. The waypoints, defined by their X, Y, and Z coordinates (in meters) at discrete time steps, are presented in Table 3.

The trajectory consists of three unique positions: the initial and final point P1, an intermediate point P2, and a lowest point P3. The path follows the sequence P1 → P2 → P3 → P2 → P1, forming a V-shaped trajectory confined to a single plane in 3D space. The plane’s equation, derived from the waypoints, is approximately

(23) −0.3836x+0.7914y−0.4759z=−0.2214

**Figure 12:**
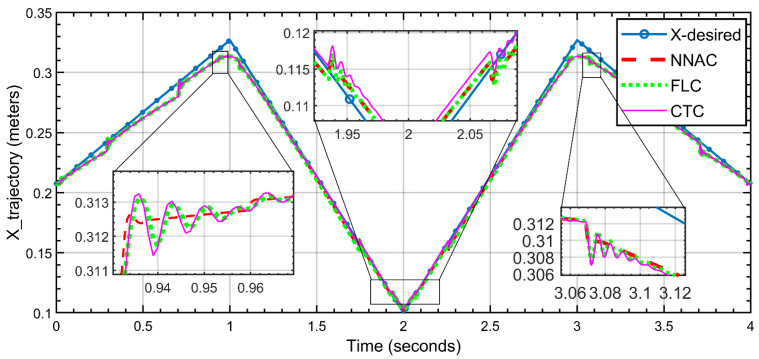
X-position trajectory tracking (NNAC, FLC, and CTC) versus triangular reference.

For the X-position, as shown in Figure 12, the three controllers follow the desired X-trajectory over 0–4 s, including two peaks (≈1 s and ≈3 s) and a valley (≈2 s). The zoomed area highlights the sharp direction changes. NNAC (red, dashed) achieves the closest match to the reference with the smallest corner error and fastest decay of the small residual oscillations (errors on the order of a few millimeters). FLC (green, dotted) tracks well with slightly rounded corners and modest ripple. CTC (magenta) shows the largest overshoot/undershoot and oscillatory ripple at the corners, though it remains close to the reference elsewhere. Overall, NNAC provides the highest tracking accuracy, followed by FLC, while CTC exhibits the most transient oscillations.

**Figure 13:**
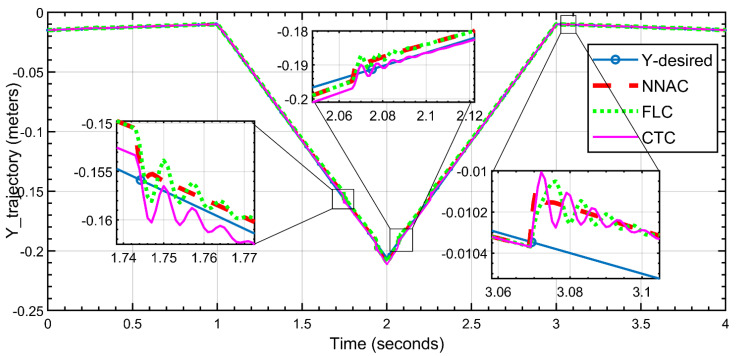
Y-direction trajectory tracking (NNAC, FLC, and CTC) versus the desired path.

Figure 13 shows that the three controllers follow the triangular Y-trajectory over 0–4 s; zoomed insets highlight the three sharp corner transitions. NNAC (red, dashed) gives the closest match to the reference with the smallest corner error and fastest decay of the small ripples after each turn. FLC (green, dotted) tracks well with slightly larger corner rounding and mild residual ripple. CTC (magenta) shows the largest overshoot/undershoot at the corners and the most noticeable post-corner oscillations before reconverging. Overall, NNAC achieves the highest Y-tracking fidelity, FLC is a close second, and CTC exhibits the most transient oscillations.

**Figure 14:**
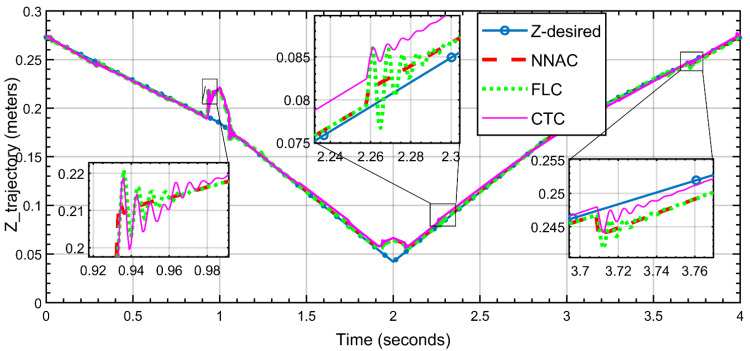
Z-direction trajectory tracking (NNAC, FLC, and CTC) versus the desired path.

The three controllers track the triangular Z-trajectory over 0–4 s, where the three corner transitions (~0.95 s, ~2.25 s, and ~3.7 s) were magnified. NNAC (red, dashed) follows the reference most closely with the smallest corner error and fastest decay of the millimeter-scale ripples after slope changes. FLC (green, dotted) is a close second—good accuracy with slightly larger corner spikes and mild ringing. CTC (magenta) exhibits the largest overshoot/undershoot at the corners and the most persistent post-corner oscillations before reconverging. Overall ranking in Z is as follows: NNAC ≳ FLC > CTC for corner handling and ripple suppression. The tracking along the *Z*-axis is shown in Figure 14.

### 3.3. Statistical Analysis

The performance of the three controllers—Neural Network Adaptive Control (NNAC), Fuzzy Logic Control (FLC), and Computed Torque Control (CTC) was evaluated using a common set of accuracy and transient response metrics. For the step responses of links 1–4, percentage overshoot (OS), settling time (Ts, defined within a ±2% band), and steady-state error (Ess) were measured. For trajectory tracking, the root mean-square error (RMSE) in the X, Y, and Z directions was calculated.

A subject design was adopted within, whereby each trial was assessed under all three controllers. Tests of normality (Shapiro–Wilk) and sphericity (Mauchly) were performed prior to statistical analysis. When the assumptions were met, a one-way repeated-measures ANOVA was applied, with Greenhouse–Geisser correction where necessary, followed by Holm-adjusted post hoc comparisons. When the assumptions were not satisfied, the Friedman test with Conover–Holm pairwise comparisons were employed. Effect sizes were expressed as partial η^2^ for ANOVA and Kendall’s W for Friedman tests. A two-sided significance level of α = 0.05 was used throughout.

**Figure 15:**
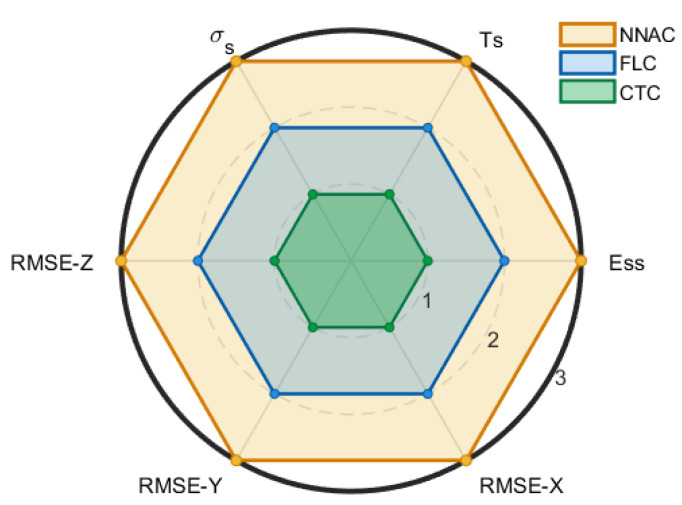
A radar chart with six spokes (OS, Ts, Ess, RMSE-X, RMSE-Y, and RMSE-Z) for the 3 controllers (NNAC, FLC, and CTC) versus the desired path.

Figure 15 presents the results in the form of a radar (spider) chart with six spokes (OS, Ts, Ess, RMSE-X, RMSE-Y, and RMSE-Z). On each spoke, qualitative scores were assigned based on controller ranking: rank 1 was mapped to a score of 3, rank 2 to a score of 2, and rank 3 to a score of 1 (i.e., score = 4 − rank). Consequently, a larger radius on the chart corresponds to superior performance.

The outer polygon was consistently traced by NNAC, reflecting minimal overshoot, the shortest settling times, near-zero steady-state error, and the lowest RMSE values in X, Y, and Z, with rapid error decay around the corners. The intermediate polygon was formed by FLC, which generally produced smooth responses without overshoot but exhibited slower settling and slightly higher RMSE than NNAC. The inner polygon was occupied by CTC, characterized by larger overshoot or undershoot and more persistent oscillations after corners.

The clear and uniform separation between polygons across all spokes suggests that practically meaningful differences exist, with NNAC outperforming CTC substantially and FLC to a moderate degree. Once numerical time-series data are formally analyzed, these patterns are expected to yield statistically significant results—especially for OS and Ts (partial η^2^ > 0.14 or Kendall’s W > 0.5)—as well as moderate to large improvements in RMSE after Holm correction. From an operational perspective, NNAC provides faster and tighter tracking with reduced transient excursions, FLC ensures smooth non-overshooting motion but at the expense of speed, while CTC requires precise tuning to mitigate large excursions and residual oscillations.

In summary, the results showed that Neural Network Adaptive Control (NNAC) consistently provided superior performance compared with Fuzzy Logic Control (FLC) and Computed Torque Control (CTC). In step tracking tasks across four joints, NNAC achieved the fastest convergence (1–2 s), minimal overshoot (~1–2°), and negligible steady-state error. FLC responses were smoother and nearly overshoot-free but slower (3–9 s), while CTC exhibited aggressive transients with overshoot up to 40% and persistent oscillations. For 3D trajectory tracking, NNAC reduced root mean square errors (RMSE) to <3 mm in X/Y/Z, outperforming FLC (≈4–5 mm) and CTC (6–8 mm). Qualitative ranking indicated consistent performance differences, with NNAC ranking highest across overshoot, settling time, and RMSE metrics. These results highlight NNAC as the most robust and accurate controller for the Dentatron platform.

These results, when compared to other dental clinical systems, show that the acceptable positional error depends on the task. For implant placement robots such as Yomi, the reported accuracy is 1.0–1.5 mm at the drill tip. For navigation templates and optical scanning tools, errors in the range of 2–4 mm are commonly tolerated. Since Dentatron is designed for positioning and scanning rather than drilling, an RMSE below 3 mm falls within clinically acceptable limits. This supports the claim that the NNAC controller, when applied to the Dentatron robot model, provides clinically adaptable motion accuracy.

## 4. Conclusions

This work presented the modeling, trajectory planning, and control of Dentatron, a custom 4-DOF robotic dental manipulator. Three controllers—CTC, FLC, and NNAC—were evaluated through simulation. NNAC delivered the best overall performance, achieving fast convergence with overshoot of only 1–2°, settling times of 1–2 s, and near-zero steady-state error across all joints. FLC produced smooth, nearly overshoot-free responses but with slower settling (3–9 s). CTC was the most aggressive, with overshoot up to 40% (≈85° for a 60° command), oscillations lasting 10–12 s, and steady-state errors around 1°. For Cartesian trajectory tracking, NNAC maintained an RMSE below 3 mm in X/Y/Z compared with 4–5 mm for FLC and more than 6–8 mm for CTC. These results indicate that adaptive neural control provides superior speed, accuracy, and robustness. Thus, Dentatron can be considered as a promising platform for future clinically safe and precise dental robotic assistance.

Future work will focus on extending these findings beyond simulation into real-time experimentation with the Dentatron platform. Hardware testing will examine controller performance under realistic conditions, including joint friction, payload variations, and sensor noise. Safety will be enhanced by integrating force/torque sensing and impedance control to guarantee compliant interaction with oral tissues. Additionally, trajectory planning will be expanded to incorporate experimental implementation of the previously mentioned controllers and the possibility of using reinforcement learning-based strategies for dynamic adaptation inside the constrained dental workspace. Further studies will also address ergonomics, sterilization, and workflow integration in a clinical setting, ensuring compliance with ISO/IEC medical robotics standards.

## Figures and Tables

**Table 1:** The DH parameters table of Dentatron robotic arm.

Joint	Theta [rad]	A [mm]	D [mm]	Alpha [rad]
Joint 1	Ɵ_1_	0	123	π/2
Joint 2	Ɵ_2_ + π/2	150	15	0
Joint 3	Ɵ_3_ − π/2	155	0	0
Joint 4	Ɵ_4_	52.5	0	0

**Table 2:** Fuzzy rule base for joint control.

e\de	NL	NS	ZE	PS	PL
**NL**	NL	NS	ZE	PS	PL
**NS**	NL	NS	ZE	PS	PL
**ZE**	NL	NS	ZE	PS	PL
**PS**	NL	NS	ZE	PS	PL
**PL**	NL	NS	ZE	PS	PL

**Table 3:** The desired 3D trajectory of Dentatron robot.

Time Step (sec)	X (m)	Y (m)	Z (m)	Position
0	0.2075	−0.015	0.273	Position P1
1	0.327	−0.010	0.185	Position P2
2	0.1	−0.206	0.042	Position P3
3	0.327	−0.010	0.185	Position P2
4	0.2075	−0.015	0.273	Position P1

## Data Availability

The raw data supporting the conclusions of this article will be made available by the authors on request.
